# Side effects during chemotherapy predict tumour response in advanced colorectal cancer

**DOI:** 10.1038/sj.bjc.6602783

**Published:** 2005-09-13

**Authors:** B Schuell, T Gruenberger, G V Kornek, N Dworan, D Depisch, F Lang, B Schneeweiss, W Scheithauer

**Affiliations:** 1 Division of Clinical Oncology, Department of Internal Medicine I, University Hospital, Waehringer Guertel 18-20, Vienna A-1090, Austria; 2 Department of Surgery, University Hospital, Waehringer Guertel 18-20, Vienna A-1090, Austria; 3 Department of Surgery, Wr.Neustadt General Hospital, Corvinusring 3-5, Wr.Neustadt A-2700, Austria; 4 Department of Surgery, Neunkirchen General Hospital, Peischinger Strasse 19, Neunkirchen A-2620, Austria; 5 Department of Internal Medicine, Kirchdorf General Hospital, Kirchdorf an der Krems A-4560, Austria

**Keywords:** colorectal cancer, side effects, chemotherapy, treatment efficacy

## Abstract

To investigate whether a relationship between chemotherapy-associated adverse events and treatment efficacy exists, we have analysed the toxicity, objective response and survival data of 303 patients with advanced colorectal cancer. Patients were divided into two groups: the first with beneficial effect (I, *n*=245), and the second with progressive disease (II, *n*=58). Differences in terms of incidence rates, type and severity of adverse events were analysed with univariate and multivariate models. The median number of side effects in group I was 6 *vs* 4 in group II (OR=1.342; *P*=0.0001). An inverse correlation between disease control and treatment tolerance was confirmed when side effects were analysed according to severity and type of treatment-associated toxicities (haematological: *P*=0.0005 *vs* nonhaematological *P*=0.0001). When median survival was analysed according to the number of adverse events, it was 10 (95% CI, 3–7), 16 (14–18), and 18 (16–20) months in case of 0–1, 2–5, and ⩾6 adverse events, respectively (*P*=0.01). In conclusion, the results of this analysis suggest that occurrence of side effects during chemotherapy in advanced colorectal cancer is an independent and reliable prognostic indicator for response and survival.

Colorectal cancer is one of the most common malignancies in the western world (Landis *et al*, 1999). Nearly one-third of all patients initially present with locally advanced, inoperable tumours and/or distant metastases (Williams *et al*, 1995; Midgley and Kerr, 1999), and approximately 50% of those having undergone potential curative surgery will ultimately develop recurrent disease. Median survival of these patients treated with best supportive care alone is approximately 6 months (Scheithauer *et al*, 1993); if treated with modern combination chemotherapy, median survival ranges from 17 to 21 months (Grothey *et al*, 2004). The effectiveness of conventional intravenous 5-fluorouracil (FU) given with or without biochemical modulators such as leucovorin (LV) or methotrexate, in fact, has been considerably improved with the recent development of a number of new, promising anticancer agents. These include the third generation 1,2-diaminocyclohexane-platinum derivate oxaliplatin, and the topoisomerase I inhibitor irinotecan. When combined with FU/LV or the oral FU prodrug capecitabine, they seem to exert a synergistic effect which results in major improvement in overall response rate and progression-free survival, occasionally in overall survival (Grothey *et al*, 2004).

Potential prognostic markers determining response and/or survival have been described in numerous studies, and include a variety of clinical and laboratory parameters. In the largest series, a multivariate analysis of 3825 patients treated with FU-based chemotherapy, performance status, the number of metastatic sites, alkaline phosphatase, and the leukocyte count were identified as the most relevant parameters (Köhne *et al*, 2002).

As it concerns the relationship between toxicity incidence and treatment efficacy, only historical data are available. As indicated by Moertel in 1969, mild to moderate toxicity as objectively calibrated by leukocytopenia seems to result in optimal response to FU as well as its deoxyriboside FUDR (Moertel and Reiemeier, 1969; Ansfield, 1975). Apart from another small patient series with rectal cancer undergoing preoperative radiochemotherapy (Dahl *et al*, 1994), to our knowledge, no other conclusive data are available about treatment tolerance as a potential prognostic marker for response despite the fact that in >80% of all patients receiving chemotherapy some kind of side effects (of course largely depending on which chemotherapy regimen is administered) will occur. Apart from use of inappropriate drug dosages in patients with impaired renal or hepatic function, presence of well-defined anticancer drug-specific risk factors such as DPD deficiency in case of FU-based chemotherapy (Diasio *et al*, 1988, 1998; Johnson *et al*, 1999; Milano *et al*, 1999), and to some extend gender (Stein *et al*, 1995; Sloan *et al*, 2002) and age >65 (Stein *et al*, 1995; Cascinu *et al*, 1996; Chiara *et al*, 1998; Popescu *et al*, 1999), the reason/mechanism is still unclear which predisposes patients to experience chemotherapy-related side effects.

As a result of uncertainties of a correlation between incidence rates and severity of side effects with the effectiveness of palliative chemotherapy in colorectal cancer, particularly as it concerns the novel more effective combination regimens, the present study was performed. In an attempt to answer this potentially important basic question, we have analysed the toxicity, objective response and survival data of 303 patients with advanced colorectal cancer who were consecutively treated in four prospective oxaliplatin- or irinotecan-based combination chemotherapy studies.

## PATIENTS AND METHODS

### Patients

A total of 303 patients with advanced colorectal cancer, who were entered in four different multicentre first- or second-line chemotherapy protocols between 1998 and 2001 were analysed (Scheithauer *et al*, 2001a, 2001b, 2001c, 2003). All patients had histologically confirmed metastatic or locally advanced/recurrent adenocarcinomas with bidimensionally measurable disease (defined as presence of at least one index lesion capable of two-dimensional measurement by computed tomography (CT) scan. Additional eligibility criteria in all four trials included age between 19 and 75 years, a World Health Organisation (WHO) performance status of two or less, and adequate bone marrow reserve, as well as renal and hepatic function. Adjuvant 5-FU-based chemotherapy and/or radiation was allowed if it was completed ⩾6 months before study entry. Within 2 weeks prior to initiating chemotherapy, all patients were assessed by physical examination, routine haematology and biochemistry analyses and CT-scans (or MRI) to define the extent of disease. Complete blood cell counts with platelet and differential counts were repeated weekly, and serum chemistries were determined at least once every course. All side effects, performance status, body weight, physical examination and subjective symptoms were recorded before each treatment course. Target lesions were reassessed by CT-scan or MRI every 8–12 weeks; objective response was evaluated according to WHO standard criteria.

### Chemotherapy

The following six treatment regimens were administered to the 303 patients, who actively participated in four different trials: The first trial was a phase II study of front-line combination chemotherapy with raltitrexed 3 mg m^−2^ and oxaliplatin 130 mg m^−2^ both administered on day 1 every 3 weeks (Scheithauer *et al*, 2001a). In the second trial, the same dose regimen was administered to patients failing prior fluoropyrimidine/LV-based chemotherapy (Scheithauer *et al*, 2001b). The third trial was a randomised multicentre phase II trial of oxaliplatin plus irinotecan *vs* raltitrexed as first-line treatment with a crossover design upon progression (Scheithauer *et al*, 2001c): patients randomised to the combination arm, were treated with oxaliplatin 85 mg m^−2^ and irinotecan 175 mg m^−2^ every 2 weeks. Patients randomised to the raltitrexed-arm received a dose of 3 mg m^−2^ given on day 1 every 3 weeks. The last trial was also a randomised multicentre phase II study of two different schedules of capecitabine plus oxaliplatin as first-line treatment in advanced colorectal cancer (Scheithauer *et al*, 2003). Half of the patients received oxaliplatin 130 mg m^−2^ on day 1 plus capecitabine 2000 mg m day on days 1–14 every 3 weeks, in the other treatment arm patients received oxaliplatin 85 mg m^−2^ on days 1 and 14 combined with capecitabine 3500 mg m^−2^ day on days 1–7 and 14–21 every 4 weeks.

### Side effects

Only chemotherapy-associated adverse reactions that occurred during the first 3 months of treatment were analysed. This was performed because (1) acute side effects generally occur within the first few courses, and (2) to avoid a possible selection bias by analysing fewer treatment courses in unresponsive patients. All except five patients received a minimum of 3 months chemotherapy (98.3%). Out of this five patients, two had a progressive disease after 2 months, two experienced severe toxicities and one patient refused further treatment after the first cycle of chemotherapy. Oxaliplatin-associated sensory neuropathy, which is cumulative in its nature, was not included in this analysis. Adverse events were graded according to WHO standard toxicity criteria. All side effects were first analysed in total, and then subdivided into haematological and nonhaematological side effects, as well as severe (grade 3/4) and minor (grade ⩽2) toxicities.

### Statistical analyses

Continuous covariates are described with mean and s.d. in the case of normally distributed data and with median, minimum and maximum for skewed covariates. Categorical variables are described with frequencies and percentages. Differences between the two interesting groups (complete remission (CR), partial remission (PR) and stable disease (SD) *vs* progressive disease (PD)) in terms of number of side effects, number of tumour sites, gender, age and chemotherapy regimen were examined with univariate and multiple logistic regression models always modelling the probability of experiencing CR; PR or SD *vs* PD. Number of side effects and all other categorisations of side effects (haematological and nonhaematological , WHO grade >2 and WHO grade ⩽2) as well as number of tumour sites are modelled as linear factors in logistic regression analyses, implying a constant odds ratio between consecutive variable values. All other scrutinised factors are taken into account as categorical variables, whereas categories male in case of gender, regimen, irinotecan plus oxaliplatin for chemotherapy, and age up to 65 years served as reference categories. All analyses were carried out using the statistical software package SAS (version 8.02, SAS Institute, Cary, NC, USA), *P*-values are two-sided and *P*<0.05 was considered statistically significant.

## RESULTS

A total of 303 patients (190 men and 113 women) who received first- or second-line chemotherapy for advanced colorectal cancer in four different clinical trials between 1998 and 2001 were included in this analysis. The pretreatment characteristics of the study population(s) which were essentially similar across the four trials are summarised in Table 1. Patients were divided into two groups: the first group (group I) had a positive treatment effect, which included complete response, partial response and SD; the second group (group II) was rated progressive during chemotherapy. As shown in Table 2, out of the 303 patients, 245 were categorised in group I, and 58 patients in group II.

Out of the entire study population only six patients had no side effects, and 297 patients had experienced at least one adverse event. The median number of side effects (haematological and nonhaematological, all WHO grades) in group I was 6 (range, 0–12) compared to 4 (range, 0–13) in case of treatment failure.

The chance to have CR, PR or SD, in fact, was noted to be increasing with an increasing number of side effects (OR=1.342, *P*<0.0001). Specifically, one additional side effect increases the odds of no PD by 34% (Table 3). The increasing incidence of side effects from PD to complete response showed a highly significant correlation (*P*<0.0001) without overlapping 95% confidence intervals from PD to SD onwards.

The observation of an inverse correlation of disease control with treatment tolerance was confirmed when side effects were analysed according to severity: in patients experiencing minor toxicities (WHO grade ⩽2; *n*=284), the median number of side effects was 3 (range, 0–10) in case of treatment efficacy, and 2 (range, 0–7) in nonresponders. The corresponding odds ratio in a univariate logistic regression model was statistically significant (OR=1.319, *P*=0.002). In patients with severe toxicities (WHO grade >2; *n*=45), responders had a median number of 2 side effects (range, 0–9), whereas nonresponder only suffered from 1 (range, 0–7; OR=1.372, *P*=0.0006).

We then divided treatment-associated side effects into haematological and nonhaematological. In all, 252 patients (83%) had haematological side effects and 289 patients (95%) had at least one nonhaematological side effect. Patients with at least SD had significantly more (*P*=0.0005) haematological side effects (median 3, range 0–4) than patients with tumour progression (median 1, range 0–4). One haematological side effect more implies a 1.472 times higher odds to experience at least SD (*P*=0.0005). The most common nonhaematological side effects (apart from the cumulative oxaliplatin-associated peripheral sensory neuropathy, which was excluded from this analysis), were diarrhoea, transient elevation of liver functional parameters and nausea/emesis. The median number of nonhaematological side effects of a responder were 4 (range, 0–9) *vs* 2 (range, 0–9) in case of treatment failure, a difference that was again statistically significant (*P*<0.0001, OR: 1.40).

We subsequently analysed the correlation of the response status and side effects according to the various treatment regimens (Table 2). Of the patients (87%) who received chemotherapy with oxaliplatin plus capecitabine were in group I (80 patients), and 13% (12 patients) had PD (group II). The median number of side effects in both groups was 6 (group I: range, 0–11; group II: range, 2–8): no difference was noted between the two groups for this particular combination regimen (*P*=0.578, OR: 1.07). In all, 82 out of 90 patients (91%) who received raltitrexed plus oxaliplatin had an objective response or SD, and eight patients had PD. The median number of side effects in group I was 5 compared with 3.5 in group II, again a difference that did not reach the level of statistical significance (*P*=0.0623, OR: 1.46). A total of 64 patients with metastatic colorectal cancer were treated with irinotecan plus oxaliplatin as first-line therapy. Of these patients, 83% were categorised in group I, and 11 patients in group II. In group I the median number of side effects was 8 as opposed to group II, where patients experienced a median number of 6 side effects (*P*=0.028, OR: 1.4). In all, 57 patients received raltitrexed as first-line chemotherapy. The median number of side effects was 5 in group I (*n*=30), and in group II (*n*=27) the median number of side effects was 3 (*P*=0.0038, OR: 1.46).

**Figure 1:**
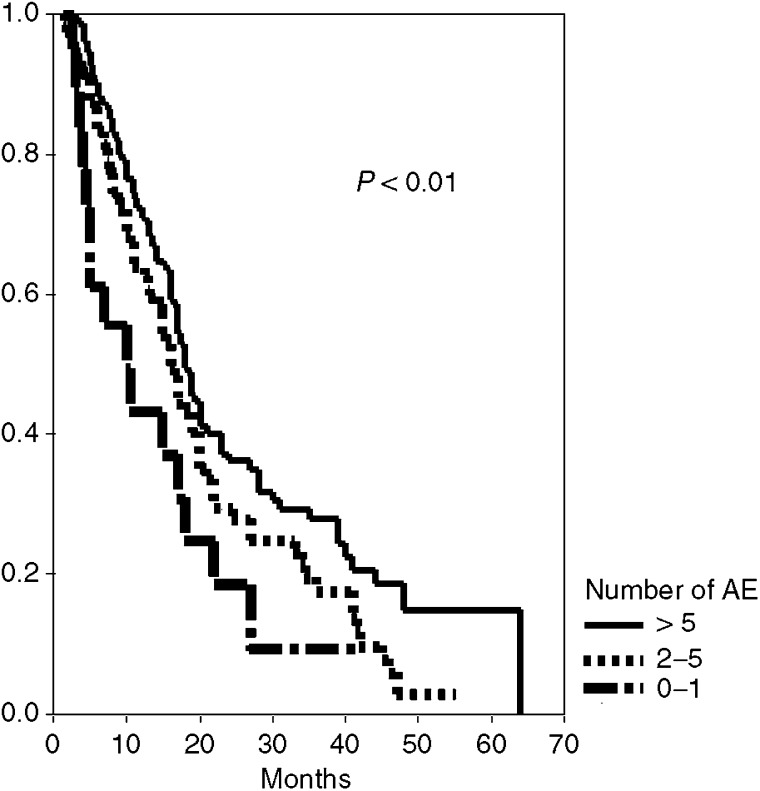
Median survival according to number of adverse events.

The median survival of all patients who had at least a stable disease was 19 months compared to 5.5 months in those with PD. When median survival was analysed according to the number of adverse events, it was 10 (95%CI, 3–7), 16 (14–18), and 18 (16–20) months in case of 0–1, 2–5 and ⩾6 AEs, respectively (log-rank test: *P*=0.01; Figure 1).

As indicated in Table 4, a multiple logistic regression model was used to assess the partial effects of number of side effects, number of tumour sites, gender, age and chemotherapy regimen in terms of treatment benefit. The number of side effects (*P*<0.0001), the number of tumour sites (*P*=0.003), and treatment with raltitrexed plus oxaliplatin (*P*=0.0081) turned out to be significant prognostic factors. As it concerns gender and age, no statistical difference was seen neither in the univariate nor in the multivariable analysis.

## DISCUSSION

Administration of the same dose regimen of a specific anticancer drug or drug combination to a population of patients is likely to result in considerable variations of toxicities ranging from no side effect to potentially lethal events (Kuilenburg *et al*, 2001; Rothenberg *et al*, 2001). In our univariate and multivariable analyses the number of side effects associated with modern combination chemotherapy in advanced colorectal cancer turned out to be a significant independent prognostic factor; one additional adverse reaction, in fact, seems to increase the odds of response or SD by 34%. Similarly, occurrence of side effects was found to correspond with an improved overall survival. The correlation between toxicity and therapeutic benefit was noted irrespective of the degree and type of toxicity, that is, nonhaematological *vs* haematological. The latter observation seems important because haematological side effects are objectively measured toxicity parameters, which are not subject to a potential reporting bias. Variations of the correlation between toxicity and therapeutic benefit between the four treatment regimens are most likely to be related to the rather small study patient populations analysed, and especially the low rate of treatment failures in case of combination chemotherapy; this hypothesis is supported by the largest correlation of adverse events and response noted in the (least effective) raltitrexed control group in one of the studies.

It seems noteworthy that the correlation between toxicity and therapeutic benefit was not affected by gender and age. Differences in reporting subjective symptoms between men and women have been described in an analysis of 2448 patients who received FU-based chemotherapy for colorectal cancer (Sloan *et al*, 2002). Women experience toxicity more frequently and with more severity than men, whereby the gender variation could be related to the methylenetetrahydrofolate reductase C677T polymorphism (Van Rijnsoever *et al*, 2002), and/or the different levels of dihydropyrimidine dehydrogenase (Diasio, 1998; Milano *et al*, 1999). The current analysis also shows no difference between sexes in objective response rate or survival. The same is true for various otherwise rather divergent data about age and toxicity (Stein *et al*, 1995; Cascinu *et al*, 1996; Chiara *et al*, 1998; Popescu *et al*, 1999). Response and survival were not affected in these studies as they were in the current study, regardless of a difference in tolerance of 5-FU-based therapy between younger and older patients.

The results of our findings with the use of modern combination chemotherapy in advanced colorectal cancer are in agreement with an analysis of the relationship between the toxicity of FU (*n*=176) and FUDR monotherapy (*n*=203) to objective response rates in a comparable study population, which was published in 1969 (Moertel and Reiemeier, 1969). Fluoropyrimidine treatment to the point of mild or moderate leukocytopenia (1500–4500) resulted in a response rate of 22.7 *vs* 8.7 and 15% in case of no (>4500) or more severe (<1500) haematotoxicity. A randomised evaluation of a planned subtoxic FU dosage conducted by the Central Oncology Group has also demonstrated a significant inferiority in response rate when compared to a dose-producing mild to moderate toxicity (Ansfield, 1975). A third analysis we could identify in terms of correlating side effects with tumour response was published by Dahl *et al* (1994). The investigators described 159 patients who were treated with preoperative radiotherapy (31.5 Gy in 18 fractions) for potentially resectable rectal adenocarcinoma. Patients were examined for a possible relationship between bowel toxicity manifested as diarrhoea and tumour size in the operative specimen as well as recurrence rate. Patients who required drugs for diarrhoea had significantly smaller tumours at surgery (2.5 *vs* 3.5 cm). Furthermore, patients without significant radiation-induced diarrhoea had more recurrences (37.5 *vs* 14.3%). The disease-specific survival rate was also significantly better (*P*=0.02) at 1.5 and 10 years in patients with diarrhoea WHO grades 3 and 4. It was therefore concluded that a correlation between bowel sensitivity and tumour sensitivity to radiation might exist.

In conclusion, the results of this analysis of over 300 patients with advanced colorectal cancer suggest that occurrence of side effects during modern combination chemotherapy in advanced colorectal cancer is an independent prognostic indicator of response. Although these data might have to be confirmed for more commonly used fluoropyrimidine combination regimens (such as FOLFOX or FOLFIRI) the correct interpretation of the present and the few other available corresponding results would already be available: as perfectly articulated by Moertel in the fluoropyrimidine monotherapy era about 35 years ago: ‘To achieve the most favourable results you must play the piper, but not too much’ (Moertel and Reiemeier, 1969). Patients should receive adequate drug dosages, which may warrant individualised dose escalations. At no price, however, treatment-associated side effects should be too severe and thus interfere with the patients' quality-of-life. As demonstrated in recent trials, dose reductions in patients experiencing severe toxicity do not lead to compromised efficacy (Cassidy *et al*, 2002; Blum *et al*, 2001).

## Figures and Tables

**Table 1:** Selected pretreatment characteristics of the analyzed study population

	**No of patients, *n* (%)**	
*Gender*		
Male	190	63%
Female	113	37%
		
*Age*		
Median	66.5	
Range	38–79	
		
*WHO performance status*		
0	138	46%
1	123	40%
2	42	14%
		
*Primary site*		
Colon	193	64%
Rectum	110	36%
		
*Sites of metastases*		
Liver	218	
Lung	75	
Abdominopelvic mass	99	
Other	118	
		
*Number of metastatic sites*		
Single	116	38%
Multiple	187	62%

**Table 2:** Descriptive statistics

	**Response to Treatment**		
	**CR+PR+SD *vs***	**PD**	
	**(*n*=245)**	**(*n*=58)**	**Total (*n*=303)**
*Gender*			
Female	88 (77.9%)	25 (22.1%)	113
Male	157 (82.6%)	33 (17.4%)	190
			
*Age*			
Up to 65 years	127 (79.4%)	33 (20.6%)	160
Older than 65 years	118 (82.5%)	25 (17.5%)	143
Mean (±s.d.)	63.12 (±9.70)	62.05 (±9.93)	62.92 (±9.74)
Min–max	37–79	38–77	37–79
			
*Number of side effects*			
Median (min–max)	6 (0–12)	4 (0–13)	6 (0–13)
			
*Number of side effects WHO-grade* < *2 (n*=*284*)			
Median (min–max)	3 (0–10)	2 (0–7)	3 (0–10)
			
*Number of side effects WHO-grade⩾2 (n*=*45*)			
Median (min–max)	2 (0–9)	1 (0–7)	2 (0–9)
			
*Number of haematological side effects (n*=*252*)			
Median (min–max)	3 (0–4)	1 (0–4)	2 (0–4)
			
*Number of non-haematological side effects (n*=*289*)			
Median (min–max)	4 (0–9)	2 (0–9)	3 (0–9)
			
*Number of tumour sites*			
Median (min–max)	2 (1–5)	2 (1–5)	2 (1–5)
			
*Chemotherapy regimen*			
Capecitabine plus oxaliplatin	80 (87.0%)	12 (13.0%)	92
Raltitrexed plus oxaliplatin	82 (91.1%)	8 (8.9%)	90
Irinotecan plus oxaliplatin	53 (82.8%)	11 (17.2%)	64
Raltitrexed	30 (52.6%)	27 (47.4%)	57

CR=complete response, PR=partial response, SD=stable disease, PD=progressive disease according to the World Health Organisation standard criteria.

**Table 3:** Univariate logistic regression analyses

	**Odds ratio**	**95% CI**	***P***
Number of side effects	1.342	1.186–1.519	<0.0001
Number of side effects WHO grade 2	1.319	1.107–1.572	0.0020
Number of side effects WHO grade >2	1.372	1.146–1.642	0.0006
Number of haematological side effects	1.472	1.183–1.832	0.0005
Number of nonhaematological side effects	1.398	1.182–1.654	<0.0001
Gender (reference group: male)	0.740	0.414–1.323	0.3099
Age (reference group: up to 65 years)	1.226	0.689–2.184	0.4880
Number of tumour sites	0.595	0.428–0.828	0.0021

**Table 4:** Multiple logistic regression analysis

	**Odds Ratio**	**95 % CI**	***P***
Number of side effects	1.349	1.167–1.559	<0.0001
Gender (reference group: male)	0.715	0.357–1.433	0.3439
Age (reference group: up to 65 years)	1.556	0.799–3.033	0.1939
Number of tumour sites	0.570	0.394–0.826	0.0030
*Chemotherapy regimen*			
Irinotecan plus oxaliplatin	1.000		
Raltitrexed plus oxaliplatin	4.271	1.457–12.521	0.0081
Raltitrexed	0.526	0.198–1.395	0.1966
Capecitabine plus oxaliplatin	1.697	0.654–4.406	0.2770
